# Proposal of a quantitative PCR-based protocol for an optimal *Pseudomonas aeruginosa* detection in patients with cystic fibrosis

**DOI:** 10.1186/1471-2180-13-143

**Published:** 2013-06-21

**Authors:** Florence Le Gall, Rozenn Le Berre, Sylvain Rosec, Jeanne Hardy, Stéphanie Gouriou, Sylvie Boisramé-Gastrin, Sophie Vallet, Gilles Rault, Christopher Payan, Geneviève Héry-Arnaud

**Affiliations:** 1 EA 3882-Laboratoire de Biodiversité et d’Ecologie Microbienne (LUBEM), SFR 148 ScInBioS, Faculté de Médecine, Université de Brest, Brest F-29200, France; 2 Département de Bactériologie-Virologie, Hygiène Hospitalière et Parasitologie-Mycologie, CHRU Brest, Brest F-29200, France; 3 Département de Médecine Interne et Pneumologie, CHRU Brest, Brest F-29200, France; 4 INSERM-CIC 0502, CHRU Brest, Brest F-29200, France; 5 Centre de Perharidy, CRCM, Roscoff F-29680, France

**Keywords:** *Pseudomonas aeruginosa*, Cystic fibrosis, qPCR, Early detection

## Abstract

**Background:**

The lung of patients with cystic fibrosis (CF) is particularly sensitive to *Pseudomonas aeruginosa*. This bacterium plays an important role in the poor outcome of CF patients. During the disease progress, first acquisition of *P. aeruginosa* is the key-step in the management of CF patients. Quantitative PCR (qPCR) offers an opportunity to detect earlier the first acquisition of *P. aeruginosa* by CF patients. Given the lack of a validated protocol, our goal was to find an optimal molecular protocol for detection of *P. aeruginosa* in CF patients.

**Methods:**

We compared two formerly described qPCR formats in early detection of *P. aeruginosa* in CF sputum samples: a qPCR targeting *opr*L gene, and a multiplex PCR targeting *gyr*B and *ecf*X genes.

**Results:**

Tested *in vitro* on a large panel of *P. aeruginosa* isolates and others gram-negative bacilli, *opr*L qPCR exhibited a better sensitivity (threshold of 10 CFU/mL *versus* 730 CFU/mL), whereas the *gyr*B*/ecf*X qPCR exhibited a better specificity (90% *versus* 73%). These results were validated *ex vivo* on 46 CF sputum samples positive for *P. aeruginosa* in culture. *Ex vivo* assays revealed that qPCR detected 100 times more bacterial cells than culture-based method did.

**Conclusion:**

Based on these results, we proposed a reference molecular protocol combining the two qPCRs, which offers a sensitivity of 100% with a threshold of 10 CFU/mL and a specificity of 100%. This combined qPCR-based protocol can be adapted and used for other future prospective studies.

## Background

*Pseudomonas aeruginosa* is the major pathogen involved in the decline of lung function in patients with cystic fibrosis (CF) [1-5]. Its presence in the lungs is associated with an increased mortality and morbidity of CF patients [6]. Early detection of this bacterium from respiratory tract is determinant because it ensures effective patient management [5,7,8]. Indeed, after intermittent colonization by different strains, once acquired, chronic *P. aeruginosa* colonization by mucoid and biofilm-growing isolates is difficult to eradicate [2,4,9,10]. Thus, the earlier the treatment toward *P. aeruginosa* onset, the higher the chance to efficiently control *P. aeruginosa *[5,7,8].

However, accurate identification of this bacterium in CF sputum by conventional microbiology techniques is known to be limited. This can be explained by a large phenotypic diversity of *P. aeruginosa* isolates recovered from CF patients such as loss of pigment production or exopolysaccharide production. Moreover, Singh et al. demonstrated that *P. aeruginosa* can form biofilms in the airways of CF patients [11]. Biofilms contain bacterial cells that are in a wide range of physiological states. One of the mechanisms contributing to this physiological heterogeneity includes the adaptation to the local environmental conditions.

For instance, bacterial cells from the deep layers of biofilm depleted of oxygen [12] can grow in anaerobic conditions. Therefore, the CF patients isolates obtained from biofilms, i.e. in anaerobic conditions, grow hardly in aerobic conditions on a conventional culture medium [13]. Another limitation of conventional culture is that *P. aeruginosa* can be easily misidentified with closely related Gram-negative bacilli in CF sputum [14-19].

The use of molecular techniques such as PCR could improve accurate identification of *P. aeruginosa *[14-19], and consequently, its early detection in CF sputum patients [20-24]. To date, there is no consensus for a universal protocol for the molecular detection of *P. aeruginosa*. Indeed, its genome is known to be highly polymorphic. Changes that can occur at the genetic level could compromise the reliability of molecular identification techniques. In particular in CF patients lungs, most of the recovered isolates are hypermutable [2,4,25,26], and show a high genetic plasticity by acquisition or loss of genes [27,28]. Since mutations or gene deletions occur on PCR target sequences, they could decrease the sensitivity of the method [29]. Moreover, horizontal genetic transfer with other bacterial species present in the CF lung niche can impact upon the specificity of the PCR [14].

In a prospective multicenter study, we aimed to assess the role of PCR for the early detection of *P. aeruginosa* in CF patients; we evaluated two qPCRs in detection of *P. aeruginosa*: a simplex qPCR targeting *opr*L gene [30], and a multiplex qPCR, targeting *gyr*B and *ecf*X genes [14]. The sensitivity and the specificity of both qPCRs were initially evaluated testing a large panel of *P. aeruginosa* isolates and closely related non-*P. aeruginosa* gram-negative bacilli isolates from CF patients. Then, the two different qPCRs ability in detection of *P. aeruginosa* were tested *ex vivo*, i.e in CF sputum samples. Finally, we were able to propose a promising reference protocol combining these two qPCRs for an optimal detection of *P. aeruginosa* in clinical setting.

## Methods

### Bacterial collection

Thirty-six *P. aeruginosa* isolates, including mucoid and non mucoid forms, were obtained from 31 sputum samples of CF patients and from 5 samples of non CF patients (blood, *n* = 1; stool, *n* = 1; urine, *n* = 1; sputum, *n* = 1; peritoneal fluid, *n* = 1), attending three French University Hospitals, the CHRU of Brest (*n* = 3), the CHU of Nantes (*n* = 26), and the GHSR of Saint Pierre, La Réunion (*n* = 2). The reference strain *P. aeruginosa* CIP 76.110 was also included in the study.

Forty-one closely related non-*P. aeruginosa* gram-negative bacillus isolates were collected, including 26 obtained from sputum samples of CF patients, and 15 from clinical samples of non CF patients (*n* = 13) or environmental samples (*n* = 2). Sixteen species were represented: *Achromobacter xylosoxidans* (*n* = 9), *P. putida* (*n* = 5), *Stenotrophomonas maltophilia* (*n* = 5), *Burkholderia cepacia* (*n* = 4), *B. multivorans* (*n* = 3), *B. gladioli* (*n* = 2), *Chryseobacterium indologenes* (*n* = 2), *Elizabethkingia meningoseptica* (*n* = 2), *P. stutzeri* (*n* = 2), *B. cenocepacia* (*n* = 1), *Flavimonas oryzihabitans* (*n* = 1), *Pandoraea pnomenusa* (*n* = 1), *P. fluorescens* (*n* = 1), *Ralstonia picketti* (*n* = 1), *Roseomonas* spp. (*n* = 1), and *Shewanella putrefaciens* (*n* = 1).

Identification of bacterial isolates was previously conducted based on phenotypical and morphological criteria (colony morphology, pigmentation, lactose fermentation, oxidase activity checked with 1% tetramethyl *p*-phenylenediamine dihydrochloride, sensitivity to antibiotics). Atypical *P. aeruginosa* isolates, for which difficulties of identification were encountered, were further analyzed with biochemical tests [API 20NE system (bioMérieux, Marcy l’Etoile, France), ID 32GN (bioMérieux)], or with the gram-negative bacillus identification card on VITEK 2 Compact (bioMéreux). All non- *P. aeruginosa* gram-negative bacillus isolates were identified by 16S rRNA gene sequencing as previously described [31].

All bacteria were stored at -80°C. Bacteria from frozen stocks were grown aerobically at 37°C for 24 to 48 hours on Muller-Hinton medium (bioMérieux).

### *P. aeruginosa* detection and quantification by sputum samples culture

#### CF patients and sample processing

Fourty-six sputa were selected in line with our study objective. These CF sputum samples have been collected from 34 patients (median age: 11 years, range: 4-29, 53% female) attending the CF center of Roscoff (France), between March 2008 and May 2012. At the time of CF patients inclusion, all of the patients were *P. aeruginosa* free for at least one year. More precisely, according to the Leeds definition [32], ten of them were never and 22 were free (Table 1). Each sputum sample was mixed with equal volume of dithiothreitol (Digesteur® Eurobio, Courtaboeuf, France) and incubated at room temperature for 30 min. For isolation of *P. aeruginosa*, liquefied sputa were immediately processed. For molecular detection of *P. aeruginosa,* two one-milliliter aliquots of every liquefied sputum were stored at -80°C.

**Table 1:** **Quantification of *Pseudomonas aeruginosa *in CF sputum samples by culture and the *opr*L qPCR and detection by the *gyr*B/*ecf *X qPCR**

**CF patient**		**CF sputum sample**	**Quantification (CFU/mL)**		**Multiplex qPCR detection (*gyr*B*/ecf*X*)***
**Anonymisation number**	*P. aeruginosa *category*		**By culture**	By *opr*L qPCR**	
003	F	1	0.0E + 00	7.5E + 00	-/-
		2	0.0E + 00	1.4E + 03	+/-
		3	2.0E + 05	2.7E + 06	+/+
004	F	4	2.0E + 03	1.2E + 05	+/+
010	F	5	1.0E + 04	9.9E + 06	+/+
012	F	6	0.0E + 00	5.0E + 01	+/-
		7	0.0E + 00	7.5E + 01	-/-
		8	0.0E + 00	2.1E + 02	-/-
		9	1.0E + 07	7.8E + 06	+/-
013	F	10	1.0E + 08	4.0E + 09	+/+
014	N	11	1.0E + 06	5.5E + 06	+/+
023	N	12	4.0E + 01	2.5E + 03	+/-
024	F	13	1.0E + 03	1.3E + 05	+/+
025	N	14	5.0E + 04	4.3E + 07	+/+
		15	1.0E + 05	3.8E + 03	+/+
026	N	16	2.0E + 06	6.7E + 07	+/+
028	F	17	1.0E + 04	1.1E + 05	+/+
030	F	18	1.0E + 03	1.3E + 04	+/+
031	N	19	1.0E + 06	1.2E + 07	+/+
		20	2.0E + 07	1.0E + 08	+/+
034	F	21	4.0E + 02	6.8E + 04	+/+
035	F	22	1.0E + 04	2.7E + 04	+/+
040	F	23	1.0E + 06	1.4E + 06	+/+
041	F	24	1.0E + 02	4.9E + 01	+/-
043	N	25	6.0E + 02	5.6E + 06	+/+
047	N	26	0.0E + 00	1.1E + 03	+/+
		27	0.0E + 00	5.3E + 03	+/+
		28	1.0E + 07	1.1E + 07	+/+
048	F	29	0.0E + 00	8.1E + 02	+/+
		30	4.0E + 01	2.5E + 02	+/+
053	F	31	1.0E + 02	5.1E + 03	+/+
054	N	32	0.0E + 00	2.3E + 01	-/-
		33	2.0E + 05	3.7E + 06	+/+
057	F	34	1.0E + 06	2.0E + 01	-/-
060	F	35	4.0E + 06	1.5E + 08	+/+
061	F	36	1.0E + 02	6.1E + 03	+/+
066	F	37	4.0E + 03	3.1E + 04	+/+
		38	1.0E + 04	9.5E + 06	+/+
070	N	39	1.0E + 06	9.0E + 07	+/+
072	F	40	4.0E + 04	7.8E + 07	+/+
076	F	41	1.0E + 03	1.5E + 04	+/+
078	F	42	1.0E + 02	2.0E + 04	+/+
202	F	43	1.0E + 05	1.7E + 05	+/-
205	F	44	1.0E + 03	3.3E + 06	+/+
220	F	45	1.0E + 06	2.3E + 08	+/+
256	N	46	1.0E + 03	3.4E + 04	+/+
mean			3.3E + 06	1.2E + 08	NA

*F: Free; N: Never (Lee et al., 2003).

**mean of quantification by *opr*L qPCR tested in duplicate.

NA: not applicable.

#### P. aeruginosa isolation

Ten *μ*l of liquefied sputum pure and diluted into 1/1000, were inoculated and incubated onto several non selective and selective media for *P. aeruginosa* isolation, including Columbia blood agar supplemented with 5% defribinated horse blood (Oxoid, Dardilly, France), Columbia chocolate agar (Oxoid), and cetrimide agar (Oxoid). All media were incubated aerobically at 37°C for five days and monitored daily. All different morphotypes of bacterial colonies were identified phenotypically with conventional screening methods (Gram coloration, oxidase test) followed by mass spectrometry identification (MicroFlex LT, Bruker Daltonics, Germany) [33,34]. Quantification was conducted based on the colony forming unit (CFU) counts and the dilution ratio of the plate.

### *P. aeruginosa* detection and quantification by quantitative PCR (qPCR)

#### DNA extraction

For each isolate of the bacterial collection, 1 ml of a 0.5 McFarland suspension was extracted. For each sputum sample, one of the two 1 ml-aliquots was treated by 5 min of sonication using a bath sonicator (Elamsonic S10, Singen, Germany). After a 10 min-centrifugation (5000 *g*), the pellet was suspended in 200 *μ*l of DNA free water. Ten *μ*l of the IC2, an internal control provided in the DICO Extra r-gene™ kit (Argène, Verniolle, France), were added in each sample and, for each batch of extraction, in 200 *μ*l of DNA free water as a negative control. DNA was extracted using the QIAamp DNA Minikit® (Qiagen, Courtaboeuf, France) according to the instructions of the manufacturer (“Tissue protocol”) with elution volumes of 100 *μ*l.

#### *opr*L qPCR

*opr*L qPCR was performed using primers *OPRL*-F and *OPRL*-R and hydrolysis probe *opr*L-MGB, previously described by Joly *et al*. [30] (Table 2). The reaction mix comprised 12.5 *μ*l of Qiagen Quantitect Probe Master Mix, 0.3 *μ*M of each primer, 0.2 *μ*M of hydrolysis probe and 4.5 *μ*l of DNA extract, and was made up to a final reaction volume of 25 *μ*l with water. A negative amplification control was used for each batch. For sputum samples, a standard curve provided a full concentration range of *P. aeruginosa* extending from 10^2^ to 10^6^ CFU/mL. Each qPCR assay was repeated twice, and the mean value of the quantification was calculated for each duplicate (Table 1). Cycling was performed on an ABI Prism 7300 Real Time PCR System (Applied Biosystem, Foster city, Californy), with an initial hold at 95°C for 15 min, followed by 50 cycles at 95°C for 15 s, and 60°C for 1 min. The *opr*L-MGB probe was labelled with carboxyfluorescein (FAM).

**Table 2:** **Primers and probes used in this study for the detection and quantification of *Pseudomonas aeruginosa***

**Name**	Sequence (5′- 3′)*	**DNA target**	**Reference**
*OPR*L-F	AACAGCGGTGCCGTTGAC	*opr*L	[30]
*OPR*L-R	GTCGGAGCTGTCGTACTCGAA	*opr*L	[30]
*opr*L-MGB	fam -TGAGCGACGAAGCC-bhq	*opr*L	[30]
*gyr*B-F	CCTGACCATCCGTCGCCACAAC	*gyr*B	[19,36]
*gyr*B-F	CGCAGCAGGATGCCGACGCC	*gyr*B	[19,36]
*gyr*B-TM	fam-CCGTGGTGGTAGACCTGTTCCCAGACC-bhq	*gyr*B	[14]
*ecf*X-F	CGCATGCCTATCAGGCGTT	*ecf*X	[14]
*ecf*X-R	GAACTGCCCAGGTGCTTGC	*ecf*X	[14]
*ecf*X-TM	yak-ATGGCGAGTTGCTGCGCTTCCT-bhq	*ecf*X	[14]

*yak = Yakima Yellow; fam = carboxyfluorescein; bhq = block hole quencher.

#### *gyr*B*/ecf*X qPCR

The *P. aeruginosa* multiplex PCR was performed using primers *ecf*X-F, *ecf*X-R, *gyr*B-F, *gyr*B-F, and hydrolysis probes *ecf*X-TM and *gyr*B-TM, previously described by Anuj in 2009 [14] (Table 2). The reaction mix comprised 12.5 *μ*l of Qiagen Quantitect Probe Master Mix, 0.4 *μ*M of each primer, 0.16 *μ*M of each hydrolysis probe, and 4.5 *μ*l of DNA extract and was made up to a final reaction volume of 25 *μ*l with free DNA water. All qPCR reaction plates contained negative amplification controls. For reaction plates containing sputum samples, a broad-range of *P. aeruginosa* concentrations from 10^2^ to 10^6^ CFU/mL was tested. Cycling was performed on an ABI Prism 7300 Real Time PCR System (Applied Biosystem), with an initial hold at 95°C for 15 min, followed by 50 cycles at 95°C for 15 s, and 60°C for 1 min. The *gyr*B-TM probe was labelled with carboxyfluorescein (FAM), whereas the *ecf*X-TM probe was labeled with a Yakima Yellow fluorophore, enabling the reaction to be distinguished using the ABI 7300 FAM and JOE detection channels, respectively. Results were analyzed by the 7300 System SDS logiciel (Applied biosystem). The *gyr*B*/ecf*X qPCR was considered positive when at least one of the two target genes was detected.

#### DICO extra r-gene amplification

Ten microliter of extracted sputum samples were distributed in 15 *μ*l of the DICO Extra r-gene premix (DP2, Argène) with 0.1 *μ*l of the HotStarTaq™ (Qiagen). The amplification program recommended by the manufacturer was applied on the automate ABI Prism 7300 Real Time PCR System (Applied Biosystem). The validation of both DNA isolation and amplification procedures, as well as the samples result interpretation, were conducted according to the instructions by Argene.

#### Determination of the lower detection threshold

To determine the lower detection threshold, six dilution ranges were realized with six different *P. aeruginosa* isolates. One range was prepared with the reference strain (CIP 76.110), two with a mucoid and a non-mucoid isolates from a sputum sample of a CF patient, and three with three isolates from three non-CF patients (urine, *n* = 1; blood, *n* = 1; stool, *n* = 1). Ten fold iterative dilutions from 0.5 McFarland calibrated *P. aeruginosa* suspensions provided a full concentration range extending from 10^0^ to 10^8^ CFU/mL. The nine dilutions of the range were tested 30 times. To determine the exact inoculum of each dilution range, a plate counting was carried out on a Mueller-Hinton medium (bioMérieux) incubated from 24 to 48 hours at 30°C. A mean of the results was calculated taking into account the sum of all assays.

#### Ethics

The Comité de Protection des Personnes Ouest VI approved the protocol. All of the patients and their relatives gave written informed consent. The collection of archival specimens was registered with the French Ministry of Research and the Agence Régionale de l’Hospitalisation, No. DC-2008-214.

## Results

### *In vitro* characteristics of the *opr*L and *gyr*B*/ecf*X qPCR

#### Sensitivity

The two qPCRs showed 100% sensitivity. At the concentration of 10^6^ CFU/mL, all the 37 *P. aeruginosa* isolates were detected by the two qPCRs. The cycle treshold (Cq) mean was 24.8 and 24/28.2 respectively for the *opr*L qPCR and the *gyr*B*/ecf*X qPCR.

#### Specificity

The specificity of the *opr*L qPCR was evaluated at 73%. At the concentration of 10^6^ CFU/mL, eleven isolates out of the 41 non-*P. aeruginosa* gram-negative bacillus isolates, corresponding to six different species, were amplified by the *opr*L qPCR. The six species responsible for cross-reactions were *A. xylosoxidans*, *B. cenocepacia*, *B. multivorans*, *E. meningoseptica*, *Roseomonas* spp., and *S. maltophilia* (Table 3). By considering the *gyr*B*/ecf*X qPCR positive when at least one of the two targeted genes was amplified, the specificity was calculated at 90%. Four out of the 41 isolates corresponding to four different species induced false positive reactions in at least one of their assays (Table 3): *C. indologenes, F. oryzihabitans*, *P. putida* and *P. stutzeri*. No species cross-reacted with both qPCRs. In this manner, combining *opr*L and *gyr*B*/ecf*X amplifications allowed achieving 100% specificity.

**Table 3:** **Bacterial species responsible for false positive amplifications with the *opr*L and *gyr*B */ecf *X qPCRs**

**Species**	**Number of isolates PCR+ / number of isolates tested**	***opr*L qPCR results**	***gyr*B */ecf *X qPCR results**
*Achromobacter xylosoxidans*	6/9	**+**	**- / -**
*Burkholderia cenocepacia*	1/1	**+**	**- / -**
*Burkholderia multivorans*	1/3	**+**	**- / -**
*Chryseobacterium indologenes*	1/2	**-**	**+ / +**
*Elizabethkingia meningoseptica*	1/2	**+**	**- / -**
*Flavimonas oryzihabitans*	1/1	**-**	**+ / +**
*Pseudomonas putida*	1/5	**-**	**- / +**
*Pseudomonas stutzeri*	1/2	**-**	**- / +**
*Roseomonas* spp.	1/1	**+**	**- / -**
*Stenotrophomonas maltophilia*	1/5	**+**	**- / -**

#### Lower detection threshold

The lower detection threshold of the *opr*L qPCR was evaluated at 10 CFU/mL. Given a positive multiplex PCR when at least one of the two probes was detected, the detection threshold of the *gyr*B/*ecf*X qPCR was evaluated at 730 CFU/mL.

### *Ex vivo* validation of the detection and quantification of *P. aeruginosa* in CF sputa by the two qPCRs

The *opr*L qPCR detected *P. aeruginosa* in all the 46 CF sputum samples. The multiplex PCR failed to detect the bacterium in five samples. The mean quantification of *P. aeruginosa* of these samples was evaluated at 67.1 CFU/mL, i.e*.* under the lower detection threshold of the *gyr*B*/ecf*X qPCR. For six of the 46 samples, only one probe (*gyr*B) was detected positive. Comparison of the results of *P. aeruginosa* quantification in CF sputum samples by culture and *opr*L qPCR is reported in Table 1. For 37 out of the 46 sputum samples tested, the quantification found by PCR is at least one log above the one found by culture. In average, for the 46 tested sputum samples, the molecular quantification of *P. aeruginosa* was two logs higher than the conventional culture quantification (1.2E + 08 CFU/mL *versus* 3.3E + 06 CFU/mL).

#### Consistency between in vitro and ex vivo experiments

The theoretical threshold calculated from *in vitro* experiments was totally consistent with the observed threshold from *ex vivo* experiments. Indeed, *opr*L qPCR assays performed *ex vivo* identified one hundred times more bacterial cells than culture-based methods did. Thus, the theoretical lower detection threshold of *opr*L qPCR of 10 CFU/mL calculated from *in vitro* cultures is equivalent to a threshold of 1E + 03 CFU/mL if applied *ex vivo*. This was verified on 9 culture-/PCR + samples for which the quantification by *opr*L qPCR retrieved a mean of quantification of 997.3 CFU/mL.

The theoretical lower detection of the multiplex qPCR was found at 7.3E + 02 CFU/mL *in vitro*. *Ex vivo*, the amplification conducted on the sputum samples showed a positive signal for at least one target (*gyr*B or *ecf*X) for all of the *P. aeruginosa*-positive sputa with quantification above 7.3E + 02 CFU/mL (n = 38). On the contrary, below 7.3E + 02 CFU/mL, the majority (5 of 8 samples) of the sputa that were *P. aeruginosa*-positive by *opr*L PCR, were *P. aeruginosa*-negative by multiplex PCR.

To conclude, the theoretical thresholds of both qPCRs were verified on the sputum samples.

## Discussion and conclusion

Several studies have suggested that qPCR is superior to culture for detecting early colonization of *P. aeruginosa* in CF sputum [20,22-24]. Today, the main goal is to have an optimal protocol as the gold standard for the molecular detection of *P. aeruginosa*. Therefore, we performed *in vitro* and *ex vivo* evaluation of two qPCRs, one targeting the *opr*L gene and the other targeting simultaneously *gyr*B and *ecf*X genes [14,30]. Numerous DNA targets have been described for the amplification of *P. aeruginosa*[15,17,19,34-36], of these three housekeeping genes, *opr*L, *gyr*B and *ecf*X have been reported to be reliable targets in the detection of *P. aeruginosa*[14,19,30,35].

The first criterion for an optimal technique in early detection of *P. aeruginosa* in CF sputum is related to the choice of the PCR format and its optimization. Today, the DNA molecules counting of a particular sequence in a complex sample can be achieved with exceptional accuracy and sensitivity sufficient to detect a single molecule [36]. As underlined by Deschagt et al. [35], the choice of PCR format may influence the performance of the molecular detection. We chose a probe-based assay, which is known to be more sensitive and specific than the SYBR Green-based qPCR [35].

The second criterion is a good sensitivity to prevent false negative results. Despite wide genetic variability of *P. aeruginosa* isolates recovered from CF patients [2,4,25-28], results of previous studies aiming at detecting *P. aeruginosa* by PCR have been encouraging. In our study, both evaluated qPCRs showed an excellent sensitivity covering all the tested panel of *P. aeruginosa* isolates. Focusing on the lower detection threshold, the difference was significant between the two qPCR assays with a detection threshold of 10 CFU/mL for the *opr*L qPCR *versus* 730 CFU/mL for the multiplex PCR. The sensitivity of the *in vitro opr*L qPCR in our study was higher than that recommended by the French guidelines, i.e. a detection threshold of 10^2^ CFU/mL for CF sputum sample [37].

The third criterion needed for early *P. aeruginosa* detection technique, in particular, for molecular one, is to have a high specificity to prevent false positive amplification. When looking at a large panel of genes described in the literature *e.g. opr*I*, opr*L*, rrl, ecf*X*, gyr*B*,* or *rrs,* specificity varied from 74% to 100% [14,17,34-36,38]. In our study, specificity of the *opr*L qPCR was evaluated at 73% *versus* 90% for the multiplex PCR. Four previous studies have tested the specificity of the *opr*L primer pairs and found different values ranging from 87% to 100% [22,34,35,38]. Again, previous studies looking at *gyr*B and *ecf*X genes found a better specificity (100%) than in our study [14,35]. Different reasons could explain these discrepancies. Firstly, our specificity could have been influenced by a larger panel of closely related non *P. aeruginosa* gram-negative bacilli (41 isolates including 16 different species). Secondly, all the bacterial isolates (except one reference strain) were recovered from clinical samples (CF or non CF) or from environmental samples. These isolates, which were recovered from CF could have undergone genetic exchange with other species in the natural CF microenvironment, especially *P. aeruginosa,* influencing the specificity of the molecular method [38]. Thus, specificity in previous studies could have been overestimated [14,34,35,38]. As highlighted by Anuj et al. [14,35], the higher specificity of our results for the multiplex PCR may be explained by the fact that we amplified at least 2 DNA targets. The use of two probes simultaneously seems to improve the specificity, providing at the same time the detection and the confirmation of the presence of *P. aeruginosa*[14,19]. Interestingly, our bacterial species that cross-reacted with the *opr*L qPCR did not do so when *opr*L qPCR was combined with the multiplex PCR thus allowing 100% specificity.

These results were successfully validated by the sputum samples of CF patients from the never or free categories according to the definition of Leeds [32]. The *ex vivo* experiments put forward a significant difference between the culture-based quantification and the qPCR-based quantification. In average, the qPCR detected 100 times more CFU of *P. aeruginosa* than the culture did.

This could be explained by different hypotheses. First, the difference in utilized sputum volumes contributes to this discrepancy. Indeed, only 10 *μ*l were cultured whereas 1 ml was extracted for the qPCR. The lowest concentration that theoretically can be detected by qPCR equals the presence of one genome (*i.e.* equivalent to one CFU) per qPCR reaction mixture. Using 1 ml of 10-fold concentrated sputum by centrifugation and extraction (elution volume of 100 *μ*l) and 4.5 *μ*l for the PCR reaction (final volume of 25 *μ*l), the detection limit of our molecular diagnosis is ≈22 CFU/mL. In comparison, the lowest concentration that theoretically can be detected by culture is 100 CFU/mL.

Second, given the phenotypic diversity of *P. aeruginosa* isolates and the large diversity of species found in pulmonary microbiota, the detection of *P. aeruginosa* by culture in CF sputum is a hard task [14-19]. Moreover, culture in aerobic conditions can fail in the detection of some isolates adapted to anaerobic conditions of the CF lung niche [13], or of non-cultivable isolates present in the bacterial biofilm [39].

Another explanation could be that qPCR detects *P. aeruginosa* DNA, *i.e.* not only live bacteria but also dead cells [40]. As CF patients are chronically treated with antibiotics, one can suppose that dead bacteria are significantly present in the pulmonary tract. In a study lead by Deschaght *et al.* in 2009, no difference in sensitivity between culture and *opr*L qPCR was found [41]. Their study was conducted on eight artificial *P. aeruginosa-*positive sputum pre-liquefied samples thus skipping the sample homogenization step, one of the cornerstones in amplification-based technique. Our *ex vivo* application of the two qPCR assays with real samples took into account the sample homogenization. It also put forward the importance of having a controlled amplification assay in particular to avoid false negatives due to inhibitors or a bad extraction. Indeed, the DNA-extraction method has been shown to be a critical step in the PCR performances [41]. In our study, we chose the DICO Extra r-gene kit, a totally artificial DNA, as internal control, which prevents from contamination during procedure handling, and allows to test extraction and amplification at the same time.

**Figure 1:**
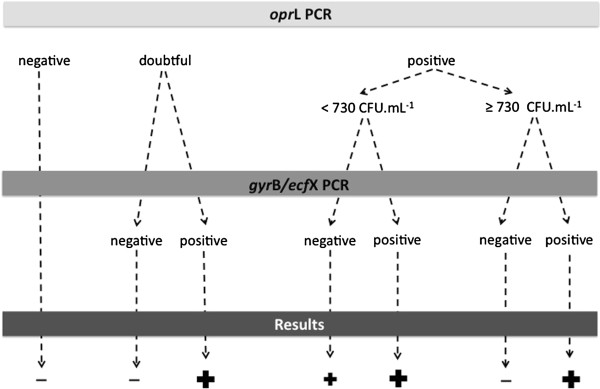
**Proposal of a molecular protocol integrating two qPCR formats (targeting *opr*L and *gyr*B/*ecf*X genes) for an early detection of *Pseudomonas aeruginosa *in sputum samples of patients with cystic fibrosis.** The *opr*L qPCR is applied in screening because of its good sensitivity. In case of a doubtful or a positive result, the *gyr*B/*ecf*X qPCR is applied in a second time. Interpretation of the *gyr*B/*ecf*X qPCR takes into account the quantification found with *opr*L qPCR. Below the detection threshold of 730 CFU/mL, the *opr*L qPCR prevails over the *gyr*B/*ecf*X qPCR. Conversely, beyond this threshold, the *gyr*B/*ecf*X qPCR prevails over the *opr*L qPCR.

Altogether, our study showed that the *opr*L qPCR offers a good sensitivity whereas the multiplex PCR offers a good specificity. Based on these data, we decided to combine these two qPCR assays and proposed a molecular protocol for an optimal detection of *P. aeruginosa* by qPCR in CF sputum as follows (Figure 1). The *opr*L qPCR can be applied in screening because of its good sensitivity. In case of a doubtful or a positive result, we can proceed to the multiplex PCR. Interpretation of the multiplex PCR takes into account the quantification found with *opr*L PCR. Below the detection threshold of 730 CFU/mL, the *opr*L qPCR prevails over the multiplex PCR. Conversely, beyond this threshold, the multiplex PCR prevails over the *opr*L qPCR. Overall, this combined molecular protocol offers a sensitivity of 100% with a threshold of 10 CFU/mL and a specificity of 100%.

This qPCR-based combined protocol can be adapted for instance in a subgroup of non-sputum producing patients and used for other future prospective studies. Indeed, the initial colonization of *P. aeruginosa* often occurs in CF patients who do not produce sputum (e.g. mainly children). This qPCR format should therefore be tested on the sample secretions routinely obtained from, e.g. deep throat swabs or endolaryngeal suction.

## Competing interests

The authors declare that they have no competing interests.

## Authors’ contributions

GHA, FLG, and RLB conceived the study and designed the experiments. FLG, GHA and RLB wrote the manuscript. FLG, SR, JH, SG and GHA performed the experiments. SBG, SV, CP and GR helped with the manuscript discussion. All authors have read and approved the final manuscript.
